# Supplementary material for: Medical honey for canine nasal intertrigo: A randomized, blinded, placebo-controlled, adaptive clinical trial to support antimicrobial stewardship in veterinary dermatology
Source: PLoS One. 2020 Aug 6;15(8):e0235689. doi: 10.1371/journal.pone.0235689 (PMC7410251; doi:10.1371/journal.pone.0235689)
Supplement: S1 Appendix — (DOCX) [file pone.0235689.s001.docx]

**Client consent form**

**Title of project:** Evaluation of Therapeutic Efficacy of Medical Grade Honey (Medihoney®) for Canine Nasal Intertrigo (Skin Fold Dermatitis)

**Objective:** Investigate the use of a medical grade honey (Medihoney®) as an alternative to topical antimicrobial therapy in the control of canine nasal intertrigo lesions.

**Manipulations :**

- **Day 1 and 22:** dermatological and general examination, bacterial culture and cytological sampling by clinician.
- **Day 1 to 21:** application of attributed product (random assignment) in the nasal fold once a day for 21 days by owner.

**Risks and disadvantages:** Medical grade honey is largely well tolerated in humans and animals. Some rare minor adverse effects exist (itching and burning sensation). Depending on random assignment, I understand that my animal might receive a placebo treatment for the duration of this study. Remaining lesions in any group will be however treated freely with a known effective therapy. I was informed of potential risks and disadvantages associated with study participation.

**Expected duration of voluntary participation and withdrawal of participation:** My animal's participation in this study is voluntary and I can withdraw it at any time without prejudice. The expected duration of the study is 21 days.

**Confidentiality:** The information collected during this study will be confidential and will not be used for other purposes. Results could however be published in a journal.

**Acceptance:** Based on the information presented in this document, I agree that [name of animal] is participating in this study.

**Date:** ____________________

**Signatures:** ________________________ ________________________

Clinician Name of pet owner

**Contact information:** Gabrielle Brosseau, DMV, IPSAV, Dermatology resident

Centre Vétérinaire DMV

800 463-8555 Dermatology service
