# Supplementary material for: Medical honey for canine nasal intertrigo: A randomized, blinded, placebo-controlled, adaptive clinical trial to support antimicrobial stewardship in veterinary dermatology
Source: PLoS One. 2020 Aug 6;15(8):e0235689. doi: 10.1371/journal.pone.0235689 (PMC7410251; doi:10.1371/journal.pone.0235689)
Supplement: S2 Appendix — (PDF) [file pone.0235689.s002.pdf]

Development of the statistical model of owner-perceived puritus VAS

The exploratory data analysis of the perceived pruritus VAS of dogs treated with medical-grade honey or placebo for 21 consecutive days revealed potential effects of the composite cytological score, composite clinical score, and differences in pruritus intensity among Pugs and Bulldogs. Consequently, we built the following five generalized linear mixed models that sequentially added the potential effect modifiers (hereafter, model names appear in **bold** characters). Of these, **Trt|Time + Cy 3**, the full model, yielded the highest goodness-of-fit and the distribution of its Pearson residuals were closest to the Normal distribution.

Model	Fixed effects	Random effects	Covariance structure
**Trt|Time 1** (basal model)	• Treatment<br>• Time<br>• Treatment×Time	• Individual dog intercept	• Pooled data
**Trt|Time 2**	• Treatment<br>• Time<br>• Treatment×Time	• Clinical score	• Pugs<br>• English or French Bulldogs
**Trt|Time + Cy 1**	• Treatment<br>• Time<br>• Treatment×Time<br>• Cytological score	• Individual dog intercept	• Pooled data
**Trt|Time + Cy 2**	• Treatment<br>• Time<br>• Treatment×Time<br>• Cytological score	• Clinical score	• Pooled data
**Trt|Time + Cy 3**	• Treatment<br>• Time<br>• Treatment×Time<br>• Cytological score	• Clinical score	• Pugs<br>• English or French Bulldogs

The following set of figures represent the estimated value of a given parameter (bold × mark) and the range of its 95% confidence interval (vertical whiskers) for each model. A reference line at Y = 0 indicates the statistical significance of the estimated parameter: those whose whiskers cross the line are non significant at α = 0.05.

Model complexity and goodness-of-fit trade-off

The Akaike information criterion (AIC) is a measure of the model's goodness-of-fit penalized for the number of estimated parameters in the model according to the formula:

$$AIC = -2 \cdot \ell + 2 \cdot d$$

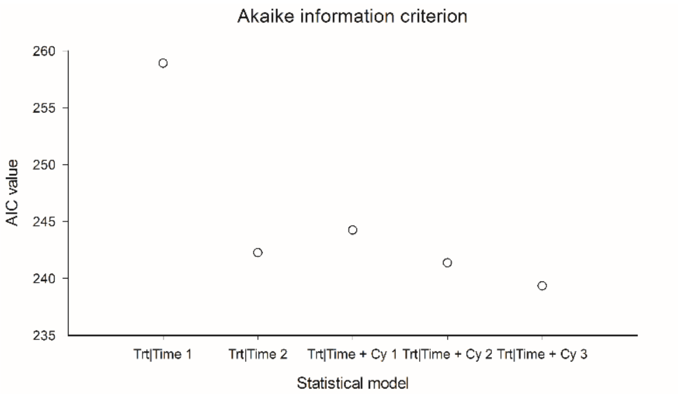

Where ℓ is the restricted maximum likelihood of the model and d is its number of estimable parameters. According to this AIC, the addition of the cytological score, clinical score or separate breed covariances to **Trt|Time 1** substantially improves the goodness-of-fit. **Trt|Time + Cy 3** has the best AIC value.

Changes in the distribution of Pearson residuals

The following table presents the distribution of conditional Pearson residuals of all models and some remarks.

Model	Histogram	Quantile-Quantile plot, normal dist.	Remarks
**Trt|Time 1**	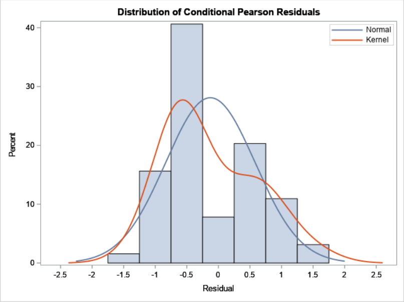	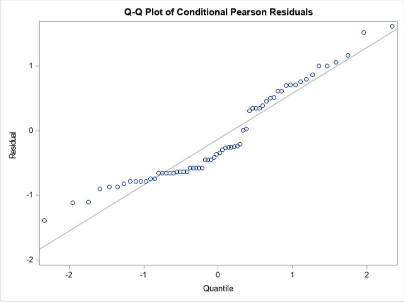	Bimodal distribution of residuals, suggesting that a clustering variable is present
**Trt|Time 2**	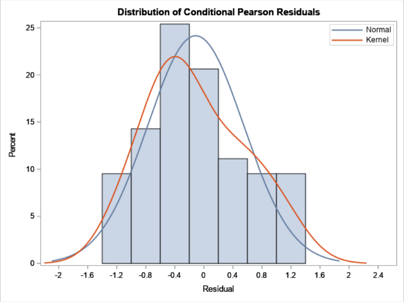	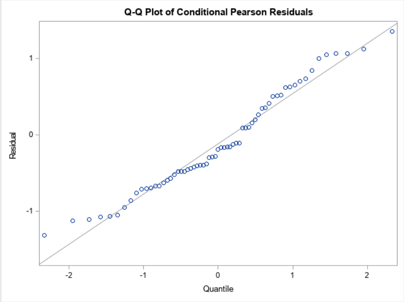	Unimodal, skewed distribution with heavy shoulders when separate covariances for Pugs and Bulldogs are used in addition to random clinical score.
**Trt|Time + Cy 1**	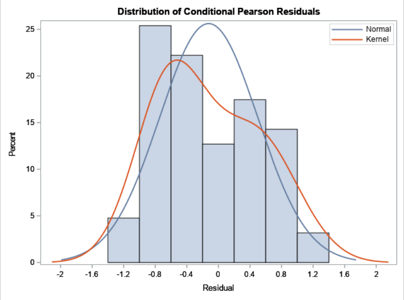	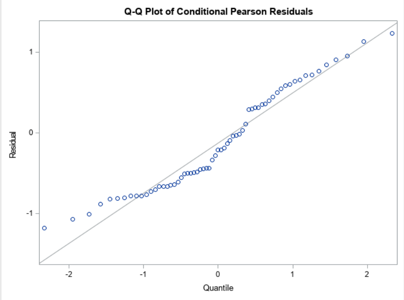	Bimodal distribution: confirms that separate-breed covariances are needed in the model.
**Trt|Time + Cy 2**	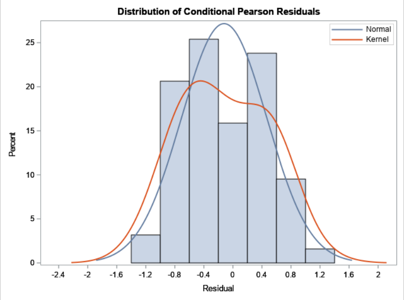	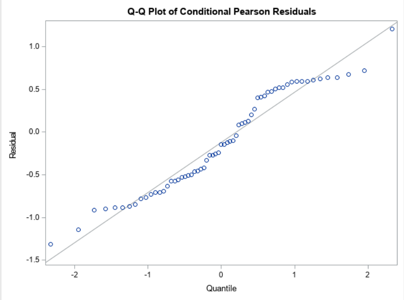	Bimodal distribution, but more symmetrical than **Trt|Time 1** and **Trt|Time + Cy 1**. Indicates that adding random clinical score only is not enough.
**Trt|Time + Cy 3**	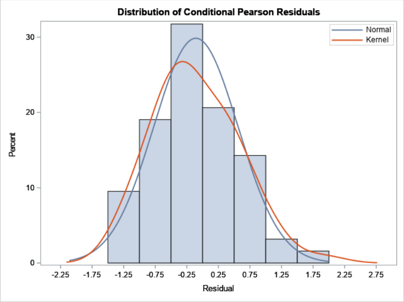	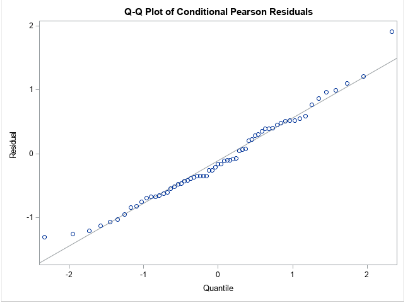	Unimodal distribution, slightly right-skewed.The kernel almost superimposes the fitted normal curve. Acceptable for further analysis.

The residuals of generalized linear mixed models must be normal-distributed for producing valid predictions. The figures of Pearson residuals histograms additionally show the fitted kernel density (red line) and normal distribution (blue line): a histogram and kernel that shape as a normal distribution are indicative of better statistical models. The quantile-quantile (Q-Q) plot allows examining how close the residuals of individual data (circles) scatter around the expected normal distribution (diagonal line): residuals that scatter closely and straightly over the diagonal from end to end are indicative of better statistical models.

Succinctly, the models using pooled covariance of individual results show a bimodal distribution, suggesting that a lurking clustering variable is present: distinguishing Pugs from Bulldog breeds corrected the bimodality. Among the tested models, **Trt|Time + Cy 3** had Pearson residuals whose distribution most closely resembled a normal distribution. Hence, we should retain this model for comparing the effects of honey and placebo on pruritus.

Changes in the estimated intercept of the linear predictor

The intercept represents the linear predictor of Day-1 VAS in dogs treated with honey.

Increasing the complexity of the statistical model did not markedly change the statistical significance of the estimated value, as shown by 95% confidence intervals that all cross the zero reference line. Yet, the introductions of the fixed-effect cytological score (**Trt|Time + Cy 1**) or of the random-effect clinical score and separate breed covariances (**Trt|Time 2**) had opposite effects on the estimated intercept.

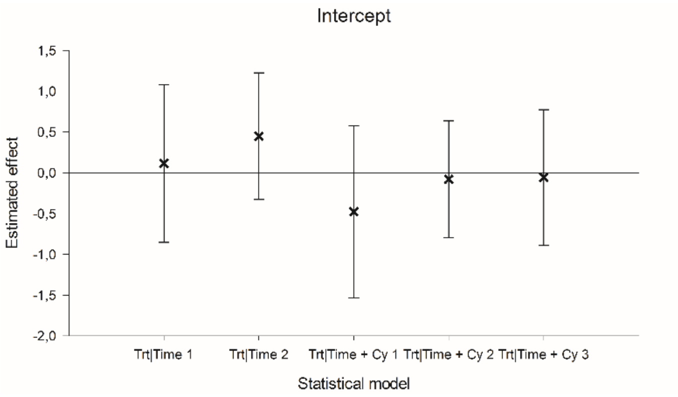

Changes in the estimated effect of Treatment on the linear predictor

Treatment represents the difference between Day-1 in dogs treated with the placebo, relative to those treated with honey.

Though none of the estimates of this parameter significantly differs from zero, increasing the complexity of the model caused a numerical decrease of the estimated difference. This decrease is associated mostly with the introduction of the fixed-effect cytological score (**Trt|Time + Cy 1**).

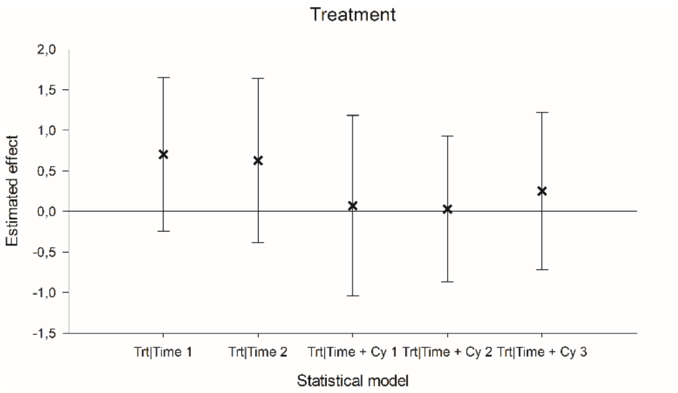

Changes in the estimated effect of Time on the linear predictor

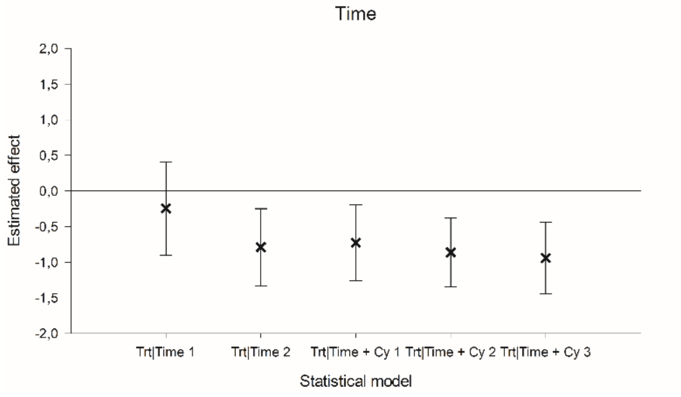

Time represents the effect size of honey at the end of the trial (at Day-22), with respect to Day-1 values.

Increasing the complexity of the model decreased the estimated effect to significantly negative values, indicating that honey had antipruritic effect. The combined addition of fixed and random-effect predictors (**Trt|Time + Cy 3**) had a slightly stronger effect than their separate additions (**Trt|Time +Cy 1** and **Trt|Time 2**), suggesting that its antipruritic effect may be masked both by the patient's clinical and cytological scores, and by differences in breed distribution among treatment groups.

Changes in the estimated effect of Treatment×Time on the linear predictor

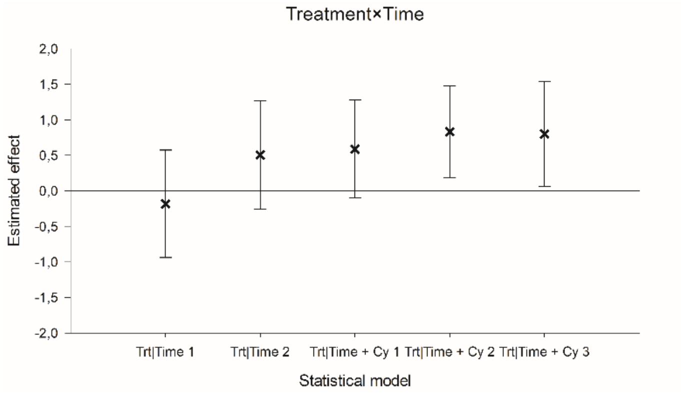

Treatment×Time represents the differential effect size of placebo at the end of the trial (at Day-22), with respect to Day-1 values, as compared to Honey.

Increasing the complexity of the model rose the estimated effect to significantly positive values, indicating that placebo had less antipruritic effect than honey. The combined addition of the fixed-effect cytological score and the random-effect of clinical score (**Trt|Time +Cy 2**) was necessary to reveal this significant effect. Differences in breed distribution among treatment groups negligibly affected the estimation of this effect (**Trt|Time + Cy 3**).

Changes in the estimated effect of combined cytological score on the linear predictor

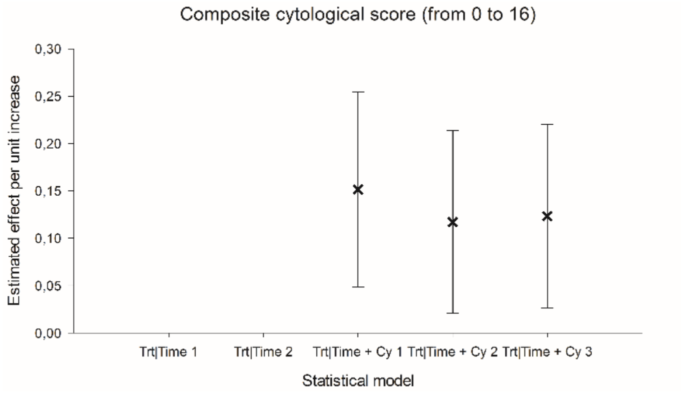

The estimated fixed effect of cytological score was positive, independently of the complexity of the model, indicating that this linear covariate had significant pruritogenic effect. Increasing the complexity of the model slightly decreased its effect, suggesting that the random effect of clinical score (**Trt|Time + Cy 2**) added to the apparent effects of cytological score on pruritus. Besides, differences in breed distribution among treatment groups apparently had little effect on the pruritogenic activity of cytological score (**Trt|Time +Cy 3**).
