# Supplementary material for: Medical honey for canine nasal intertrigo: A randomized, blinded, placebo-controlled, adaptive clinical trial to support antimicrobial stewardship in veterinary dermatology
Source: PLoS One. 2020 Aug 6;15(8):e0235689. doi: 10.1371/journal.pone.0235689 (PMC7410251; doi:10.1371/journal.pone.0235689)
Supplement: S2 Table — Count, cell frequency in a two-way table; Fisher, bilateral Fisher exact test; Mean, arithmetic mean; SD, standard deviation; Student, bilateral Student t-test for two independent samples with identical variances; Trt, treatment. Count variables: number of dogs with matching data (e.g. Pugs vs. French or English Bulldogs, neutered vs. sexually intact dogs, carriers vs. non-carriers of a given microbial isolate at the site of intertrigo). Abbreviations of microbial isolates: E.coli, Escherichia coli; E.faecalis, Enterococcus faecalis; Hafnia sp., Hafnia species; Klebsiella sp., Klebsiella species; K.variicola, Klebsiella variicola; L.adecarb., Leclercia adecarboxylata; M.pachyderm., Malassezia pachydermatis; MRSP, methicillin-resistant Staphylococcus pseudintermedius; MSSP, methicillin-sensitive Staphylococcus pseudintermedius; P.aeruginosa, Pseudomonas aeruginosa; P.mirabilis, Proteus mirabilis; S.aureus, Staphylococcus aureus; S.canis, Streptococcus canis; S.schleferii, Stahpylococcus schleferii; W.confusa, Weissella confusa. (DOCX) [file pone.0235689.s006.docx]

| **Variable** | **Key**  **Figure** | **Placebo**  **(16 dogs)** | **Honey**  **(13 dogs)** | **Statistical**  **Test** | **Raw**  **P-value** |
| --- | --- | --- | --- | --- | --- |
| Age | Mean (SD) | 5.24 (2.55) | 4.53 (2.89) | Student | 0.4861 |
| Pug | Count | 8 | 6 | Fisher | 1.0000 |
| Male | Count | 10 | 9 | Fisher | 1.0000 |
| Neutered | Count | 15 | 10 | Fisher | 0.2994 |
| MSSP | Count | 8 | 11 | Fisher | 0.1142 |
| MRSP | Count | 2 | 0 | Fisher | 0.4877 |
| S.aureus | Count | 0 | 1 | Fisher | 0.4483 |
| S.schleferii | Count | 3 | 0 | Fisher | 0.2315 |
| S.canis | Count | 8 | 7 | Fisher | 1.0000 |
| K.variicola | Count | 1 | 0 | Fisher | 1.0000 |
| Klebsiella sp. | Count | 0 | 1 | Fisher | 0.4483 |
| E.coli | Count | 5 | 2 | Fisher | 0.4100 |
| P.aeruginosa | Count | 6 | 1 | Fisher | 0.0927 |
| E.faecalis | Count | 0 | 1 | Fisher | 0.4483 |
| P.mirabilis | Count | 2 | 0 | Fisher | 0.4877 |
| Normal flora | Count | 1 | 0 | Fisher | 1.0000 |
| M.pachyderm. | Count | 1 | 0 | Fisher | 1.0000 |
| Hafnia | Count | 0 | 0 | Fisher | 1.0000 |
| W.confusa | Count | 0 | 0 | Fisher | 1.0000 |
| L.adecarb. | Count | 0 | 1 | Fisher | 0.4483 |
