# Supplementary material for: Medical honey for canine nasal intertrigo: A randomized, blinded, placebo-controlled, adaptive clinical trial to support antimicrobial stewardship in veterinary dermatology
Source: PLoS One. 2020 Aug 6;15(8):e0235689. doi: 10.1371/journal.pone.0235689 (PMC7410251; doi:10.1371/journal.pone.0235689)
Supplement: S3 Table — BH-adj., Benjamini-Hochberg adjustment for controlling the false discovery rate; Raw, uncorrected probability value; Trt, treatment. Abbreviations of microbial isolates: E.coli, Escherichia coli; E.faecalis, Enterococcus faecalis; Hafnia sp., Hafnia species; K.variicola, Klebsiella variicola; Klebsiella sp., Klebsiella species; L.adecarb., Leclercia adecarboxylata; M.pachyderm., Malassezia pachydermatis; MRSP, methicillin-resistant Staphylococcus pseudintermedius; MSSP, methicillin-sensitive Staphylococcus pseudintermedius; P.aeruginosa, Pseudomonas aeruginosa; P.mirabilis, Proteus mirabilis; S.aureus, Staphylococcus aureus; S.canis, Streptococcus canis; S.schleferii, Stahpylococcus schleferii; W.confusa, Weissella confusa. (DOCX) [file pone.0235689.s007.docx]

| **Microbial isolate** | **Placebo**  **(16 dogs)** | **Honey**  **(13 dogs)** | **Raw**  **P-value** | **BH-adj.**  **P-value** |
| --- | --- | --- | --- | --- |
| MSSP | 8 | 12 | 0.0020 | 0.3196 |
| MRSP | 1 | 0 | 1.0000 | 1.0000 |
| S.aureus | 0 | 1 | 0.4483 | 1.0000 |
| S.schleferii | 4 | 0 | 0.1067 | 0.8539 |
| S.canis | 5 | 6 | 0.4657 | 1.0000 |
| K.variicola | 1 | 0 | 1.0000 | 1.0000 |
| Klebsiella sp. | 0 | 0 | 1.0000 | 1.0000 |
| E.coli | 4 | 4 | 1.0000 | 1.0000 |
| P.aeruginosa | 3 | 1 | 0.6059 | 1.0000 |
| E.faecalis | 1 | 1 | 1.0000 | 1.0000 |
| P.mirabilis | 3 | 1 | 0.6059 | 1.0000 |
| Normal flora | 0 | 0 | 1.0000 | 1.0000 |
| M.pachyderm. | 1 | 0 | 1.0000 | 1.0000 |
| Hafnia | 1 | 0 | 1.0000 | 1.0000 |
| W.confusa | 1 | 0 | 1.0000 | 1.0000 |
| L.adecarb. | 0 | 0 | 1.0000 | 1.0000 |
