# Supplementary material for: Medical honey for canine nasal intertrigo: A randomized, blinded, placebo-controlled, adaptive clinical trial to support antimicrobial stewardship in veterinary dermatology
Source: PLoS One. 2020 Aug 6;15(8):e0235689. doi: 10.1371/journal.pone.0235689 (PMC7410251; doi:10.1371/journal.pone.0235689)
Supplement: Attachment — Submitted filename: Response to reviewers.docx [file pone.0235689.s008.docx]

# Response to reviewers, Manuscript PONE-D-20-01412

## Journal requirements

| **Serial #** | **Request / Comment** | **Response** |
| --- | --- | --- |
| 1 | Ensure that your manuscript meets PLOS ONE's style requirements, including those for file naming. See PLOS ONE style templates (the URL appear as a footnote).^[1]^ | Unfortunately, the URL links to the style templates provided in the decision email do not work (i.e. error 404), but we found them at the URL provided in the following footnotes.^[2],^^[3]^  All tables were formatted according to the manuscript style template.  The legends of the supporting information files were added according to the manuscript style template, and the sequence of supporting files modified accordingly to their in-text citation order.  A “Conclusions” section was added to the revised manuscript, according to the template. In addition, the word “captions” was deleted from the section title on p.42/l. Fig 5 was duplicated after Fig 4 (it was already available as “striking image” at the end of the composite file),  The prefix “full title:” was deleted from page 1/line 3 of the original manuscript, the short title was deleted from p.1/l.6, and the initials (GB) were moved from the end of p.1/l.16 to the end of p.1/l.17, according to the relevant template. |
| 2 | Report additional details about the 'MediHoney' product used in this study: chemical characterization or measures of purity performed, additional processing steps taken other than irradiation, e.g. heating. | At the time of planning this study, we were unaware of the growing issue of Manuka honey counterfeiting and assumed that Medihoney™ 100% Pure Antibacterial Honey respects its label claim (i.e., is pure *Leptospermum* honey produced in New Zealand).  Unfortunately, we are unable to perform the requested purity characterizations because our institutions have suspended all non-essential laboratory activities due to the COVID-19 pandemic, we have no product left from the lot number used in the trial, and doubt the reliability of extrapolating purity assessment from a current lot to earlier ones.  We applied no heating or any other physical or chemical process on the tested honey and always kept it at room temperature, including during the transfer from the original to the blind-coded tubes.  Therefore, we added the following sentence to our study limitations (lines 572-5 of the revised manuscript), and referred to recent studies focusing the authentication tests for Manuka honey:  “**Fourth, we did not have access to analytical techniques to ensure the purity of the tested lot of medical-grade honey [72, 73], but dog owners using the product likewise rely on the reputation and internal quality control procedures of the product manufacturer and distributors.**”  Added references (lines 876-83 of the revised manuscript)  **72. Burns DT, Dillon A, Warren J, Walker MJ. A Critical Review of the Factors Available for the Identification and Determination of Manuka Honey. Food Anal Method. 2018;11(6):1561-7. doi: 10.1007/s12161-018-1154-9.**  **73. McDonald CM, Keeling SE, Brewer MJ, Hathaway SC. Using chemical and DNA marker analysis to authenticate a high-value food, manuka honey. NPJ Sci Food. 2018;2:9. Epub 2018/05/22. doi: 10.1038/s41538-018-0016-6.** |
| 3 | Provide the data in replacement of the mention “data not shown” PLOS does not permit references to inaccessible data. | The sentence in p.18/l.354-6 of the original manuscript was rephrased as follows (lines 316-19 of the revised manuscript):  “**None of Treatment, Time, and Treatment×Time significantly influenced the composite microbial abundance score (Table 3), and none of the recorded patient characteristics improved the model’s goodness of fit to the data**”  Table 3 of statistical analysis of the composite microbial score was added: see the section “Additional changes” below. |
| 4 | Upload a copy of Figure 5, to which you refer in your text on page 24. | Fig 5 was already uploaded as the “striking image” of the original manuscript. It is now added in duplicate in the revised manuscript, following Fig 4. See the section “Duplicated Fig. 5” at the end of this document. |

## Reply to Reviewer #1 comments

| **Serial #** | **Comment / Request** | **Response** |
| --- | --- | --- |
| 1 | Tables in the main text are difficult to follow and main conclusions are not clear.  Some of the statistical tables perhaps are better suited for supplementary).  It would be of benefit to simplify these tables and include in the legend what each of the testing scores are measuring/how they were determined. | Table 3 of the original manuscript has been deleted and is now available as S1 Table.  The following “conclusions” were added to improve the clarity of the manuscript, according to the PLoS manuscript template (lines 579-86 of the revised manuscript):  “**Based on the results presented in Tables 3-5 and 7 and Figs 1-3, we cannot recommend the use of medical grade Manuka honey as an alternative to topical antimicrobials in the control of canine nasal intertrigo, as its therapeutic efficacy was inferior to our placebo hydrogel. The alleviation of pruritus (Table 6 and Fig 4) appears as a short-term gain for the well-being of both the dog and its owner, but its inability to resolve the microbial, cytological and clinical components of the disease may outweigh this benefit. Our adaptive trial design methodology opens new grounds for testing the efficacy of veterinary therapies.**”  A description of how the scores were determined was added to the legend of their corresponding table, e.g. (lines 327-9 of the revised manuscript):  “**The composite microbial abundance scores were obtained for each dog at Day-1 and 22 by adding the growth scores (i.e. from 0 = no growth, to 4 = visible growth beyond the 2^nd^ quadrant) of all lesion-site organisms cultured on Mueller-Hinton agar plates using the 4-quadrant streaking method.**” |
| 2 | Is the sensitivity of the bacterial isolates/species identified in the study to honey known / can they be determined? It could be of value to determine the concentration of honey required to inhibit the growth of these species (e.g. MIC testing) as sub-inhibitory concentrations can often boost the growth of some bacteria. | Unfortunately, we have neither the expertise nor the access to a microbiology research laboratory, and potential collaborations in this area are unlikely to be established in the near future, as the COVID-19 pandemic has forced the hibernation of all non-essential laboratory activities in our respective institutions. In addition, we foresee some challenges in interpreting the minimal inhibitory concentration of a mixture of compounds that may exert synergistic antimicrobial activity, but whose respective proportions may vary non-negligibly from lot to lot. Nonetheless, we have found the following studies that may address your concern, at least in part:  Uri M, et al. A pilot study comparing in vitro efficacy of topical preparations against veterinary pathogens. Vet Dermatol. 2016 Jun;27(3):152-e39. PMID: 27109449.  This study determined the minimal bactericidal or fungicidal concentrations (MBC/MFC) of a commercial brand of medical-grade Manuka honey and other types of antiseptics for commonly isolated canine skin pathogens: 3 *Staph. pseudintermdius*, 2 *E. coli*, 1 *P. aeruginosa* and 1 *Malassezia pachydermatitis* isolate, some sensitive and other resistant to antimicrobials. The isolate suspensions were exposed for 3 or 10 min to honey at concentrations ranging from 15.26 to 100 g/L, and then plated on an agar suitable for the tested pathogen. The MBC/MFC of Manuka honey could be determined for none of the tested organisms and exposure times. The colony forming units of MSSP, MRSP and *Malassezia pachydermatitis* at the higher exposure levels decreased with contact time*.* The short contact time of the pathogens to the Manuka honey likely explain these results.  Grego E, et al. Evaluation of antimicrobial activity of Italian honey for wound healing application in veterinary medicine. Schweiz Arch Tierheilkd. 2016 Jul;158(7):521-7. PMID: 27504886.  This study used the broth microdilution method for determining the MIC and MBC of Medihoney®, the medical-grade Manuka honey used in our study, for 7 pathogens isolated from canine wound infections, including *Staph. aureus* (either sensitive or MRSA)*, Enterococcus fecalis, Escherichia coli,* ESBL-producing *E. coli, P. mirabilis and P. aeruginosa*. Medihoney® displayed the highest antibacterial activity, with MIC ranging between 3.1% and 25% (v/v) and MBC values identical to the MIC of the tested pathogens. The pathogens were exposed to Medihoney® for 24h.  Tramuta C, et al. Antibacterial activities of Manuka and Honeydew honey-based membranes against bacteria that cause wound infections in animals. Schweiz Arch Tierheilkd. 2017 Feb;159(2):117-121. PMID: 28174146.  This study is a follow-up of the previous one (Grego et al., 2016), in which a time-kill method was used for determining the survival over 24h of 4 canine wound pathogens, including methicillin-resistant *Staph. pseudintermedius*, ESBL-producing *E. coli*, *P. aeruginosa, and P. mirabilis,* on honey-based pectin membranes containing either Medihoney® or honeydew (1:1 v/v). The Gram-negative pathogens were under the recovery limit after 3h incubation on the Medihoney®-HBM, and only 1h incubation time was needed for obtaining a similar result for *S. pseudintermedius*.  Cremers N, Belas A, Santos Costa S, Couto I, de Rooster H, Pomba C. In vitro antimicrobial efficacy of two medical grade honey formulations against common high-risk meticillin-resistant staphylococci and Pseudomonas spp. pathogens. Vet Dermatol. 2020 Apr;31(2):90-96. PMID: 31808237.  This study used the broth dilution method for determining the MIC of two commercial brands of medical-grade Manuka honey against *Staphylococcus* spp. (n=11) and *Pseudomonas* spp (n=11) isolates, including *S. aureus, S. epidermitis, S. pseudintermedius, S. schleiferi, P. aeruginosa* and *P. fluorescens*. For the first formulation (40% of Manuka honey), bacteriostatic effects were achieved at 10% dilution for all staphylococci, except for a MRSP strain (20%). A MBC (minimal bactericidal concentration) of 10% was observed for most of the staphylococci isolates tested. This honey had a MIC and MBC of 20% for all *Pseudomonas* strains.  For the second formulation (80% of Manuka honey), a bacteriostatic effect was observed at 10% for most staphylococci isolates. Few isolates displayed a MIC between 2.5% and 5% and one MRSP strain presented a MIC of 40%. The staphylococci MBC values ranged from 5% to 40%. This formulation had a MIC and MBC of 40% against almost all *Pseudomonas* strains. |
| 3. | The authors have identified the limitations of the study thoroughly. In line with my comment above, if the bacteria tested are not sensitive to honey, the dose (concentration/amount) of honey applied in the study may not be sufficient to inhibit bacterial growth and/or reapplication may have been necessary sooner - the methods state that a thin layer was applied. As microbial load is one of the foundations of the scoring used in the study, this could affect the final conclusions being drawn. | The above-listed studies of the antimicrobial activity of Manuka honey have shown that the most prevalent veterinary skin pathogens are sensitive to Manuka honey. We agree that reapplying the tested products may increase the antimicrobial activity of the honey, however, the repeated cleaning, drying and reapplication of the product could cause irritation of the treated zone. In addition, increasing the frequency of manipulations often results in a decreased compliance of the owners. Therefore, it is difficult to predict whether a more frequent product application would indeed provide better outcomes. |
| 4. | Figure 5 is missing in the file. | Figure 5 was uploaded as the “striking image” in the original manuscript submission. We have duplicated its addition in the revised composite file |

## Response to Reviewer #2 comments

| **Serial #** | **Comment / Request** | **Action** |
| --- | --- | --- |
| 1a | Well conducted and written study worth publishing, although it is very elaborate and could be abbreviated some. | We have shorten the manuscript by 352 words (7 in the introduction, 200 in the materials and methods, 116 in the results, and 54 in the discussion) to bring it down to 5029 words, including the conclusions and acknowledgements. We additionally moved Table 3 of Day-1 exploratory data analysis to the Supplemental data section. See the “Additional changes” section below for more details. |
| 1b | However, the main problem in understanding is that honey seems to improve pruritus versus placebo improves cytology and clinical signs. Then it is stated that increased cytology scores went along with increased pruritus scores. Makes sense as well, however, then I would assume that honey also should also improve cytology. Did I understand this wrong? This needs to be clearer in the manuscript. | We may not perfectly understand this concern, and apologize if the following explanation misses the point.  We built our statistical models as a causal relationship of a set of predictor variables that determine the variation of the outcome variable. This differs from a correlation test that measures the linear association between two variables, which are not necessarily tied by a causal relationship (e.g., the variables are independent from one another, but both are modulated by an unaccounted predictor). In contrast, the causal relationship is directional (i.e., the predictor variable acts on the outcome variable but a defined mechanism must be identified for the opposite be true).  In Table 4 of our manuscript, which estimates the fixed effects of Time, Treatment, Treatment×Time, and a dichotomized microbial score on the cytological score, we disclosed that honey did not significantly influence the variation of cytological scores. We tested this with the Time predictor variable: Day-22 cytological scores in honey-treated dogs did not significantly differ from Day-1 values (Time = 0.06683±0.1398; P=0.2075). This result is logical because the cytology score is the sum of the abundance grades of cocci, rods, yeasts and inflammatory in a smear of material collected from lesion site, and Table 3 reveals that honey had no significant effect on the microbial abundance score (Time: -0.08581±0.1692; P = 0.3522).  Since these inflammatory and microbial cells can produce pruritogenic substances, it is logical to find in Table 6 that the cytological score acts as a significant predictor of the variation of pruritus scores (Cy_Score: 0.1234±0.04697; P = 0.0147). Cy_Score acts as a covariate in this model, implying that its effect applies to the dogs in both treatment groups and on both measuring occasions. Therefore, the following phrase (underlined) was added to the manuscript to clarify the issue (line 417 of the revised manuscript):  “**Finally, the cytological score increased the perceived intensity of dog’s pruritus for all treatment groups and assessment times (P=0.015).**”  Despite being unable to curb the microbial and cytological scores, honey achieved a significant antipruritic effect (Time: -0.9410±0.2443; P = 0.0018). In the discussion (lines 479-487), we indicated that the antipruritic effects of honey were expected and suggested the roles of some flavonoids (chrysin and galangin) in inhibiting the inflammatory components of pruritus.  In other words, honey could not decrease the abundances of microbial and inflammatory cells, but successfully blocked their secreted substances that trigger the pruritus. |
| 2 | Line 4: I am not sure if the reference to antibiotic stewardship is needed in the title, may be the Editor should comment on that. | We understand this concern but respectfully suggest keeping it in the title, because ours is one of the few trials that tested an alternative treatment method to save the available antimicrobials for more severe infections in our private veterinary dermatology service. This was not a contract research project sponsored by the manufacturers of the tested product. |
| 3 | Line 24: "... medical-grade ..." | Done (see line 22 of the revised manuscript) |
| 4 | Line 28: "... the test or the placebo product ..." | Test was replaced by “honey”, and placebo written with lowercase (line 26-7 of the revised manuscript) |
| 5 | Line 37: "... P=0.0443). ..." | Done (line 35 of the revised manuscript) |
| 6 | Line 40: "... (cytological score ..." | Done (line 38 of the revised manuscript) |
| 7 | Line 61: A reference should be inserted for the atopic dogs | We deleted the mention to atopic dogs from line 59 of the revised manuscript, because the article that explicitly linked the two conditions (in the abstract) did not convincingly show an association between canine atopic dermatitis and intertrigo (in fact, the author posited this association but did not care to verify its existence through of scientific research). See:  Scarff D, Intertrigo: the hidden face of canine atopic dermatitis. Companion Anim 2016; 21: 384-90. |
| 8 | Line 99: "... inert treatment arm, ..." | Done (line 97 of the revised manuscript) |
| 9 | Line 102: "... of this study was to ..." | Done (line 100 of the revised manuscript) |
| 10 | Line 123: "... size was either ..." | Done (line 119 of the revised manuscript) |
| 11 | Line 172: "... were combined into a ..." | Deleted because of its redundancy with respect to the sentence starting on line 215 of the revised manuscript. |
| 12 | Line 236: "... products was the time ..." | Done (line 225 of the revised manuscript) |
| 13 | Line 242: "...respectively were H0 ..." | Done (line 230 of the revised manuscript) |
| 14 | Line 247: "... trial had a maximum ..." | Rephrased (line 234 of the revised manuscript) |
| 15 | Line 249: "... were blinded, the hypotheses were .... was assumed ..." | Done (lines 235 and 237 of the revised manuscript) |
| 16 | Line 254: "... determined ..." | Done (line 241 of the revised manuscript) |
| 17 | Line 293: "... received the placebo and the other 16 received the medical honey. Of these ..." | Done (line 278 of the revised manuscript) |
| 18 | Line 296: "... given placebo was discarded ..." | Done (line 280 of the revised manuscript) |
| 19 | Line 299: "... treated with placebo and 13 with honey ..." | Done (lines (283-4 of the revised manuscript) |
| 20 | Line 307: "... two with placebo and one with honey ..." | Rephrased as follows: “two placebos and one honey” (line 293 of the revised manuscript) |
| 21 | Line 340: "... in the treatment group and of 2 isolates at both times in the placebo group ..." | Reformulated as follows: “… in placebo-treated dogs, and of 2 isolates at both times in honey-treated dogs.” (lines 308-9 of the revised manuscript) |
| 22 | Line 369: "... additions of breed or sex to the ..." | Mention to these variables was deleted to shorten the manuscript (lines 343 of the revised manuscript) |
| 23 | Line 374: "... for placebo treated dogs than for honey-treated dogs ..." | Done (lines 347 of the revised manuscript) |
| 24 | Line 377 and 378, 408 and 409, 437 and 438, 485 and 486, 488, 498: Ditto | Done (multiple lines in the results and discussion sections of the manuscript) |
| 25 | Line 438 and 439: Do the authors mean that the pruritus increased with the cytological score (and thus correlated to the cytology)? But that does not fit the discussion and results. Or that the pruritus decreased with increased cytology? That would not make sense .... Please rephrase. | See response to comment 1b. We hope that our explanation clarified that the causal relationships of cytological score (predictor A) and treatment with honey (predictor B) on the pruritus score (outcome) cannot imply a correlation between the two predictors. |
| 26 | Line 459-465: Is this needed or could you just delete this? | We deleted some redundant information from this section, but kept the rest because it discloses the statistical testing of the specific abilities of each treatment at improving the intertrigo scores. This differs from Tables 3-6, which measure the extent to which the treatments differ from one another. For instance, one treatment may be inferior to the other (as tested in Tables 3-6), yet both treatments may be therapeutically beneficial. The results presented in Table 7 rule out this possibility.  Therefore, we modified the title of the subsection (lines 438-9) as follows:  “**Hommel-adjusted testing of the therapeutic benefit of tested articles**”  Lines 440-3 have been rephrased as follows:  “**Unilateral statistical testing (i.e., targeting a score reduction) of the therapeutic efficacies of the placebo and medical honey are summarized in Table 7, and representative patients illustrated in Fig 5. Only placebo achieved significant cytological and clinical benefits, and only honey significantly alleviated the pruritus VAS.**”  To lighten table 7, the column “Sim. Adj. P” was deleted, and the “final inferential analysis” and reference list were shortened because mention to simulation-based adjustment of P-values was no longer needed (lines 273-4 and 786 of the revised manuscript). |

## Additional changes

| **Serial #** | **Change (line of revised manuscript)** | **Motivation** |
| --- | --- | --- |
| 1 | Line 38: added statistic in abstract | The estimated effect and associated P-value of the composite cytological score on the owner-assessed pruritus severity was missing. See Table 6. |
| 2 | Line 40: corrected statistic in abstract | The estimated effect and P-value included in the original abstract was the one of placebo, not that of honey. See Table 6. |
| 3 | Line 114: Delete study duration | Redundant with respect to line 126 |
| 4 | Line 146: Honey brand name corrected | Change made according to product packaging |
| 5 | Line 164: Mention to Idexx laboratory deleted | Redundant with respect to line 203 |
| 6 | Lines 168-80: Simplification of the paragraphs describing intra-trial and post-trial treatment of dogs | Decrease word count |
| 7 | Lines 247-73: Simplification of the paragraphs describing the inferential statistical analyses | Decrease word count |
| 8 | Lines 296-306: simplification of the description of the blinded exploratory data analysis | Decrease word count |
| 9 | Line 306: Table 3 of the original manuscript was moved to supplemental data | Improve the readability of the manuscript |
| 10 | Lines 308-22: Simplification of the description of microbial culture results | Decrease word count |
| 11 | Line 323: New table 3 added (see below) | Requested by PLoS Editorial Office |
| 12 | Lines 329-31: Description of the composite microbial score added to the legend of Table 3. | Stand-alone Table, as requested by Reviewer #1 (serial # 1) |
| 13 | Lines 334-6: Description of the composite microbial score added to the legend of Fig 1 | Stand-alone Figure, as requested by Reviewer #1 (serial # 1) |
| 14 | Lines 345-54: Simplification of the description of cytological score results | Decrease word count |
| 15 | Lines 361-3: Description of the composite cytological score added to the legend of Table 4. | Stand-alone Table, as requested by Reviewer #1 (serial # 1) |
| 16 | Lines 369-70: Description of the composite cytological score added to the legend of Fig 2. | Stand-alone Figure, as requested by Reviewer #1 (serial # 1) |
| 17 | Lines 378-86: Simplification of the description of clinical score results | Decrease word count |
| 18 | Lines 392-4: Description of the composite clinical score added to the legend of Table 5. | Stand-alone Table, as requested by Reviewer #1 (serial # 1) |
| 19 | Lines 399-401: Description of the composite clinical score added to the legend of Fig 3. | Stand-alone Figure, as requested by Reviewer #1 (serial # 1) |
| 20 | Lines 408-12: Simplification of the description of pruritus score results | Decrease word count |
| 21 | Lines 426-7: Description of the pruritus score added to the legend of Table 6. | Stand-alone Table, as requested by Reviewer #1 (serial # 1) |
| 22 | Line 433: Description of the pruritus score added to the legend of Fig 4. | Stand-alone Figure, as requested by Reviewer #1 (serial # 1) |
| 23 | Lines 457-9: Legend added for Fig. 5 | Stand-alone Figure, as requested by Reviewer #1 (serial # 1) |
| 24 | Discussion section: simplification of some paragraphs | Decrease word count |
| 25 | References: some references were added or deleted | As a consequence of reviewer’s requests |

### Newly added Table 3

Table 3. Estimated coefficient of the linear predictor of Poisson-distributed composite microbial abundance scores, standard errors, boundaries of the 95% confidence intervals, and results of the type-3 statistical testing of fixed effects.

| **Effect** | **Level** | **Estimate** | **SE** | **95% C.I. Bounds** | | **Type-3 Test** | | |
| --- | --- | --- | --- | --- | --- | --- | --- | --- |
|  |  |  |  | **Lower** | **Upper** | **Den. DF** | **F Statistic** | **P-value** |
| **Intercept** |  | 1.5652 | 0.1848 | 1.1892 | 1.9413 |  |  |  |
| **Day** | 22 | -0.08581 | 0.1692 | -0.4325 | 0.2609 | 28 | 0.89 | 0.3522 |
| **Trt** | A | 0.1473 | 0.2249 | -0.3133 | 0.6080 | 28 | 0.44 | 0.5140 |
| **Trt*Day** | A-22 | -0.02568 | 0.2092 | -0.4543 | 0.4029 | 28 | 0.02 | 0.9032 |

### Duplicated Fig. 5

1. <http://www.plosone.org/attachments/PLOSOne_formatting_sample_main_body.pdf>
   and [http://www.plosone.org/attachments/PLOSOne_formatting_sample_title_authors_affiliations.pdf](https://can01.safelinks.protection.outlook.com/?url=http%3A%2F%2Fwww.plosone.org%2Fattachments%2FPLOSOne_formatting_sample_title_authors_affiliations.pdf&data=01%7C01%7Cgbrosseau%40centredmv.com%7C6548929d2bbd4c6cee6908d7d15d4ef4%7C340025f283ca484e9155a3ab3fb7b708%7C0&sdata=ANtaH89ZFem3DXGa9DpUPxxpjo8Lu8Uh4nORNT6j0m0%3D&reserved=0)
2. <https://journals.plos.org/plosone/s/file?id=4497/Main%20Body%20-%20ONE%20Formatting.pdf>
3. <https://journals.plos.org/plosone/s/file?id=ba62/PLOSOne_formatting_sample_title_authors_affiliations.pdf>
