# Supplementary material for: Medical honey for canine nasal intertrigo: A randomized, blinded, placebo-controlled, adaptive clinical trial to support antimicrobial stewardship in veterinary dermatology
Source: PLoS One. 2020 Aug 6;15(8):e0235689. doi: 10.1371/journal.pone.0235689 (PMC7410251; doi:10.1371/journal.pone.0235689)
Supplement: Attachment — Submitted filename: Response to reviewers-R2.docx [file pone.0235689.s009.docx]

# Response to reviewers, Manuscript PONE-D-20-01412R1

## Reviewer #1:

The manuscript reads with much more clarity in this revision. Some minor suggestions:

| **Serial #** | **Comment / Request** | **Response** |
| --- | --- | --- |
| 1a | Tables are very heavy on statistical analyses and values, some of which are (not) explained in the results text very well (…). As an example, Table 2 could have a simpler heading (Blinded interim analyses to accept or reject null hypothesis), and the information in the current title could be used in the table legend. (…) These are just suggestions and not essential to change for the manuscript to be accepted - if it is common in the field to report the results as such, then this suggestion can be overlooked.  I had similar confusion with Table 4 - the title could be simplified to what is in the results text (Estimated fixed effects of treatment, time, and treatmend x time on composite cytology score); and then state what is it we are looking at i.e. what values are most important in this table to make your conclusions. It would be helpful to state which value is important and what it is testing - this is done very well for the latter tables in the manuscript (eg. Table 5 where the text specifies the result and conclusion, then reports the Cy_score or trt x time values). | Thank you for your comments.  As suggested, we added explanations that specify the results and conclusions in the results text for the tables that were lacking it (Tables 3 and 7), as it was presented for Table 5.  As suggested, we simplified the titles of Tables 2 to 7, and moved the previous titles of the tables to their respective legends. |
| 1b | Alternatively, it might be useful to state what the hypothesis/null hypothesis is in the table legend for ease of readability. | We added a recall of the hypothesis and null hypothesis in the Table 2 legend to ease readability, as suggested.  (Line 301-6): “**Legend: This table presents the sample size, actual Fisher information value and result of the blinded first interim statistical analysis testing the null hypothesis of equal Treatment×Time means on the linear predictor of the intertrigo composite clinical score in the placebo and honey-treated dogs, I.e., H0: µ(A) - µ(B) = 0. As a recall, we hypothesized that medical-grade Manuka honey would be safe and clinically superior to placebo at treating nasal intertrigo. The expected amount of Fisher information that the trial would gather for testing the null hypothesis was 23.0 for a maximum sampling size of 86.6 dogs (Table 1).**”  Please note that newly added text is underlined, and highlighted text fulfills your specific requirements. |
| 1c | The values in the tables could be reduced to 2 significant figures to simplify the table further. | In order to further lighten the tables, we have rounded their values to 1, 2 or 3 decimals, depending on the integer and estimated standard error. |
| 1d | When reporting the results here for Table 2, the value in the text refers to -0.370 but this doesn't match values in the table. | We developed the explanation prior to Table 2 to clarify the values:  (Line 292-8): “**Table 2 reveals that, after collecting 33.5% of the maximum sample size (i.e., 29/86.6; Table 1), the first interim analysis gathered sufficient Fisher information to reject the null hypothesis of equal mean effects of the tested articles: Z = -2.16, outside the -1.97 lower decision boundary. The estimated effect of Treatment×Time on the linear predictor of clinical score was -0.37 with 95% confidence interval = [-0.71, -0.04], which negative values indicate that the placebo decreased the clinical score more effectively than honey (P = 0.03).”**  Please note that newly added text is underlined, and highlighted text fulfills your specific requirements. |
| 2 | Figure legends should specify what panel A and B are (placebo and honey). | Thank you for this suggestion, we made the changes. |
| 3 | My comments regarding the sensitivity of the bacterial isolates/species in this study to honey have been addressed and I agree that the microbiological assays do not necessarily have to be conducted. However, I would recommend including the point in the discussion - perhaps in the limitations section. E.g. it would be useful to state that the microbes commonly associated with this condition have been reported as being sensitive to honey (you already have the references showing this in the author comments), and perhaps the antimicrobial effect of honey was not observed here due to the amount used for treatment or frequency of application. It could be a contributor to why the honey was not effective overall since the microbial load is one of the scoring parameters used. There are studies showing that too low a concentration of honey can promote bacterial growth, particularly biofilms that could be cited to make this point. | We added the following sentences to our study limitations:  (Line 639): “ **Sixth, since the most prevalent canine skin pathogens are sensitive to Manuka honey in vitro [74-76], and sub-inhibitory in vitro exposure to this product increases the tolerance, viability and biofilm production of *P. multocida* [27], the lack of antimicrobial effect recorded during this trial may suggest that the tested honey was scantly or too sparsely applied. However, the dog owners received instruction to apply the product per manufacturer’s instructions, and the repeated cleaning, drying and product reapplication may both irritate the treated zone and decrease owner compliance.**”  Please note that newly added text is underlined, and highlighted text fulfills your specific requirements. |

## Reviewer #2

Thank you for addressing most of my previous comments. There are a couple more, but they are fixed easily enough. However, there is still a misleading tenor about the pruritus that is not supported by looking at the dogs from a clinical (and not statistical point of view (see below). Please find my comments below.

| **Serial #** | **Comment / Request** | **Response** |
| --- | --- | --- |
| 1 | Line 37: “… clinical score (cytological …” | Thank you for this correction. We changed it in the manuscript as follows:  (Line 37): “**For every unit increase in cytological score, the linear predictor of clinical score increased by (...),**” |
| 2 | Line 58: “… brachycephalic dogs such as pugs …” | Thank you for this correction. We changed it in the manuscript. |
| 3 | Line 65: “… to preserve their efficacy …” | Thank you for this correction. We changed it in the manuscript. |
| 4 | Line 165: “… An animal health technician randomized each dog’s treatment with a coin toss and kept …” | Thank you for this correction. We changed it in the manuscript. |
| 5 | Line 427: When looking at the dogs’ VAS values in Fig. 4, 4 dogs improved in the honey group and one dog got worse, and four dogs in the placebo group improved and none got worse, even the slope in the dogs improving was similar, and you still state that pruritus improved in the treatment group. (“Fig 4 depicts the effects of treatment on the integer of visual analogue scale values of owner-perceived pruritus in dogs: honey decreased the perceived intensity of pruritus more consistently than placebo.”). I still don’t understand this and even if it is statistically significant, it certainly is clinically absolutely irrelevant in my opinion and the statement that pruritus was decreased more consistently with honey treatment is misleading. This could be clarified in the paragraph starting at line 478, where a statement about the difference between statistically significant and clinically significant could be mentioned, which would completely alleviate my concerns, as the authors have drawn the right conclusions from the study data and the study was well done. | Thank you for clarifying your concern. We already agree that the effect of the treatments on VAS likely was of lower importance as compared to the cytological and clinical scores, as stated in the conclusion of the 1^st^ revision of our manuscript. Moreover, after reading your highlighted phrase about Fig. 4, we realized that for its last part, we inadvertently drifted to the findings of Table 6 without indicating it explicitly. Thank you for pointing this out.  However, your conclusion that the effect of honey on the pruritus VAS was “clinically absolutely irrelevant” grounds from an interpretation of Fig. 4 that neglects the other determinants accounted for in our statistical model. This concern is associated with: (i) several predictors of the statistical model of VAS cannot be illustrated adequately in Fig 4, (ii) the overlay of individual VAS score variations, and (iii) reducing the score variations to qualitative outcomes (i.e., pruritus improved, remained unchanged, or worsened) in function of time and treatment caused loss of information. We will develop only the first, most important:  In addition to treatment, time and their interaction, the generalized linear mixed-effect model of pruritus VAS included the fixed effect of cytological score at day-1 and 22, the random clinical score at day-1 and 22, and separate covariances for Pugs and Bulldogs. Our statistical model abated the masking effects of these predictors, allowing the effects of Time and Time×Treatment on VAS to show statistical significance, as shown below, but we did not find a convenient way to illustrate this in Fig. 4.  The following figures depict the change of effect size and statistical significance of estimated fixed effects of Time and Treatment×Time as a function of the predictors included in the statistical model. The Y axis represents the estimated effect size, the X axis represents the statistical models, the crosshair symbols are the estimated effect sizes and the whiskers represent the 95% confidence intervals of estimated effects. When the 95% C.I. whiskers vertically cross 0, the estimated effect is not significant at the 0.05 α-level. The depicted models sequentially add the above-listed fixed- and random-effect predictors:   a. Model “Trt\|Time 1” contains only the fixed effects of Time, Treatment, Treatment×Time, and random intercept for each dog. b. Model “Trt\|Time 2” is identical to the previous model but replaces the random intercept of dogs with their random clinical scores at Day-1 and 22 and separate covariances for Pugs and Bulldogs. c. Model “Trt\|Time + Cy 1” has the same fixed and random effects than model Trt\|Time 1 above, but additionally includes the fixed effect of cytological score. d. Model “Trt\|Time + Cy 2” is identical to the previous model but replaces the random intercept of dogs with their clinical scores. e. Finally, model “Trt\|Time + Cy 3” is identical to the previous but additionally includes separate covariances for Pugs and Bulldogs.   This first figure shows that the effect of Time (i.e., the effect of honey on pruritus score) is masked mainly by the effect of cytological score (shown by model Trt\|Time + Cy 1), its negative value indicates an antipruritic effect. The top whiskers below zero indicate a statistical significance that is reinforced by the additions of the random effect of clinical score and separate covariances for Pugs and Bulldogs. This second figure shows that the effect of the Treatment×Time interaction (i.e. the differential effect of placebo on pruritus, relative to honey) is masked mainly by the combined effects of clinical score (shown by model Trt\|Time 2) and cytological score (shown by model Trt\|Time + Cy 2). It reaches statistical significance only after both these predictors are added to the model and remains significant after adding the covariances for Pugs and Bulldogs. The positive effect size value indicates that placebo has significantly less antipruritic effect as compared to honey, and Table 7 later shows that its ability at improving pruritus is not significantly better than null.  Among these, model Trt\|Time + Cy 3 is the only valid one because its distribution of Pearson residuals resembles a normal distribution.  Hence, the difference among treatment groups may seem trivial, but we cannot safely conclude that it is clinically irrelevant without further experimental evidence.  We decided nonetheless to present Fig 4 in the manuscript for the sake of transparency and homogeneity with respect to the reporting of the other three scores. Another, more didactic reason to present Fig 4 to the readers is to illustrate the importance of exploratory data analyses in every statistical analysis: figures of raw data may not reveal the whole picture when predictors have opposite effects on the outcome variable. When not used in the graphs, the hidden predictors can confound the relationships of the predictors graphed with the outcome variable.  Therefore, we have modified our manuscript as follows:  (Line 451-7): “**Fig 4 depicts the variation of owner-perceived pruritus scores in dogs in each treatment group: although honey significantly decreased the perceived intensity of pruritus more effectively than placebo (Table 6), this difference was unapparent in the graphs. Noteworthy, the cytological score, clinical score and breed are hidden determinants of the time-course of pruritus VAS illustrated in Fig 4. An appraisal of their effects on the significance of the fixed effects of the model is available in S7 Appendix.**”  And  (Line 508-19): “**However, Fig. 4 failed to provide visual confirmation of this finding because the plotted data had no correction for the masking effects of breed, cytological score and clinical score tackled by the statistical model. We identified these effect modifiers during the exploratory data analysis, confirming the critical importance of this initial stage of statistical analysis (S7 Appendix). Study designs using stiffer inclusion criteria or stratified randomization that limit the variation or distribute more evenly the dog breeds and their initial microbial, cytological and clinical scores across study arms prior to treatment onset, may better determine whether this antipruritic effect not only has statistical significance but also clinical significance. However, these alternative designs imply additional patient visits, lower patient inclusion rates, and a delayed treatment onset with the test articles that potentially hinder the well-being and disease remission of patients.**”  Please note that newly added text was underlined, the highlighted text fulfills your requested change, and the paragraph’s text following the ellipsis sign in parentheses (…) is identical to our previously revised manuscript. |

## Reviewer #3

I will focus on methods and reporting. Methods are appropriate but unnecessarily complex in my view. Overall, a good manuscript, however, with carefully conducted analyses.

| **Serial #** | **Comment / Request** | **Response** |
| --- | --- | --- |
| 1 | Major #1: There is no clear discussion on power calculations. How did the authors arrived at the final sample? Create a section labelled "power calculations" and expand there. If you didn't perform any, discuss as a major limitation. | Power calculations typically are part of a study report when the trial fails to reject the null hypothesis, which was not our case. Of the 4 scores that were subject to statistical analysis, only the Treatment×Time effect on our composite microbial abundance score did not show significance at α=0.05, but this was not the pivotal outcome variable (lines 226-30). From the estimated Treatment×Time effect presented in Table 3 (following line 338), 1395 patients per treatment group are needed to reach significance at α=0.05 and β=0.10 in a classic clinical trial. We developed the first limitation of our previously revised manuscript as follows:  (Line 596-600): “**First, the number of dogs included was the minimum required to reject our null hypothesis of equal treatment effects on the clinical score. Differences in cytological and pruritus scores additionally were significant, but the estimated difference in microbial burden score would require 1395 dogs/group to reach significance at α = 0.05 and β = 0.10. (…)**”  Please note that newly added text was underlined, the highlighted text fulfills your requested change, and the paragraph’s text following the ellipsis sign in parentheses (…) is identical to our previously revised manuscript.  With respect to sample size calculations, we stated in line 114-5 that “This study was (…) adaptive group sequential trial,” i.e., not a classic clinical trial.  In a classic clinical trial, sample size calculations are performed based on prior information of the effect sizes for each study group, enabling the estimation of a difference among groups and associated variance. But this often is an historical comparison of studies whose methodologies may differ, so the estimation is prone to error. Hence, calculated sample sizes may be either too large or too small, especially if there is no prior data or poor-quality data with which estimate the effect sizes and variabilities of each treatment.  This precisely is the case with canine intertrigo. Indeed, a PubMed search of the MeSH terms “intertrigo/veterinary” OR “intertrigo/drug therapy” yield respectively 2 and 86 entries: the two veterinary entries were general reviews on bacterial dermatitis published more than 40 years ago, and the 86 therapeutic entries relate to human or rodent models of human intertrigo, which therapeutic efficacy results cannot be inferred safely to canine intertrigo because of obvious differences in skin morphology, physiology, microbiology and other predisposing factors. Despite the paucity of references, intertrigo is a common problem in brachycephalic dogs and a frequent motive for case presentation in veterinary practice.  As mentioned at the beginning of our discussion (lines 491-4 of the newly revised manuscript), to circumvent this lack of reliable data with which calculate our sample sizes, we applied the adaptive group-sequential trial methodology. As opposed to a classical trial, the adaptive trial uses the accumulating data gathered during the trial in a predetermined number of interim analyses to precise the final sampling size or early stop the trial if sufficient evidence has been gathered, as disclosed in lines 115-23, lines 226-42 and Table 1 of the materials and methods section. The sample sizes at each interim and the final analysis derive from the values of minimally relevant difference in clinical effects, α and β error probabilities, and a conservative estimate of the standard deviation of the target difference provided in lines 232-40 of the materials and methods. Table 1 specifies the statistical boundaries for both the acceptation and the rejection of the tested null hypothesis. |
| 2 | Major #2: The analyses are quite complicated because of the repeated design. Why was this chosen? What is the justification. I can understand it when we are dealing with cancer patients and we need an early decision to save lives, but in the context of canine nasal intertrigo seems like overkill. | We have chosen a trial design with pre- and post-treatment examinations because intertrigo is a periodically recurring condition whose presentation, duration and severity vary among patients (line 61-2), at variance with a chronic disease that has a stable, easily subtractable baseline value.  In the introduction section of the manuscript, we have explained that canine nasal intertrigo is a relatively benign skin infection that, unless permanently removing the predisposing condition (which is not always possible), requires periodical treatment with antibiotics or antiseptics (lines62-3). The latter are drivers of antimicrobial resistance selection (lines 64-5) that can spread in both dogs and in-contact humans (lines 53-5). Because antimicrobial resistance is a global emergency that hampers the ability of both physicians and veterinarians to combat infectious disease (lines 48-9), we looked for effective alternatives to antimicrobials (lines 82-3).  Among them, medical-grade Manuka honey seemed promising because of its antimicrobial activity and several other beneficial effects (lines 84-92), but quality evidence of its efficacy in companion animals is lacking (lines 78-80 and 95-100). Therefore, we decided to generate our own data to guide future therapeutic decisions in our private veterinary dermatology referral service but had a very limited budget to accomplish this research (i.e., Canadian $3000). Hence, optimizing the sampling size was crucial to our ability at conducting this trial.  As effect sizes and variabilities were available for none of the possible treatment products that may be used for the therapy of canine intertrigo, and the placebo effect of these products is seldom measured, we concluded that a classical trial design was inappropriate for our study goals and constraints. Hence, we believe that the adaptive group-sequential trial method was the most suitable alternative. |
| 3 | Major #3: Table 1 on page 12 is not informative for most people so you need to clarify that "actual" is, and the tole of the rejection bounds (explain they are z-values in the text as well). | In this and Table 2, the information level refers to the Fisher information of the study, which is 1/variance of the estimated Treatment×Time effect on the linear predictor of clinical score. The column headline and table legend have been modified accordingly.  We added the following legend to Table 1:  (Lines 247-52): **“Legend: This table presents the expected increases in Fisher information, I.e. the reciprocal of variance, of the estimated Treatment×Time effect on the linear predictor of composite clinical score, as a function of the sample size at different trial stages (first interim, second interim and final statistical analysis). In addition, the table presents the statistical boundaries for rejection or acceptation of the null hypothesis of equal mean effects at each stage. The higher the value of Fisher information is, the higher the precision of the estimated effect size.”** |
| 4 | Minor #1: Double blinded. I am not sure how this applies in this case. I can understand why the dogs were blinded but how were the owners blinded? I would imagine it would be obvious if you were using manuka honey or hydrogel? or am I missing something here? | Thank you for sharing your concern. In fact, the double blinding in this study refers to the dog owners and the investigators, not the dogs. Since owners are the ones that apply the test products on their dogs and record the perceived pruritus with a VAS, they must be blinded of the treatment received. The placebo hydrogel contained ethyl phenylacetate to provide a honey-like fragrance and was packed into plastic tubes identical to the ones filled with honey (lines 151-6 of the materials and methods). |
| 5 | (Minor #2) some language corrections are needed. | We corrected additional language errors:   1. Line 23: ‘’ (…) flora of intertrigo, and recorded (…)’’ was changed for ‘’ flora of intertrigo and recorded’’ 2. Line 84: ‘’standardized’’ was changed for ‘’standard’’ conditions 3. Line 90: ‘’cicatrisation’’ was changed for ‘’cicatrization’’ 4. Around line 142: ‘’ glucocorticoid’’ was changed to ‘’ glucocorticoids’’ 5. Line 220: ‘’ evenly-spaced intervals’’ was changed for ‘’ evenly spaced intervals’’ 6. Line 312: ‘’Dogs (…) ranged between 8 months and 12 years of age’’ was changed for ‘’Dogs (…) ranged from 8 months to 12 years of age’’ 7. Line 573: “lipfold” was changed with “lip fold”. |
